# Immunogenicity of Co-Administered Omicron BA.4/BA.5 Bivalent COVID-19 and Quadrivalent Seasonal Influenza Vaccines in Israel during the 2022–2023 Winter Season

**DOI:** 10.3390/vaccines11101624

**Published:** 2023-10-22

**Authors:** Stephen Moss, Menucha Jurkowicz, Ital Nemet, Nofar Atari, Limor Kliker, Bayan Abd-Elkader, Tal Gonen, Emily Toth Martin, Yaniv Lustig, Gili Regev-Yochay, Michal Mandelboim

**Affiliations:** 1 Department of Epidemiology, University of Michigan School of Public Health, Ann Arbor, MI 48109, USA; stcmoss@umich.edu (S.M.);; 2 Department of Epidemiology and Preventive Medicine, School of Public Health, Faculty of Medicine, Tel Aviv University, Tel Aviv 5265601, Israel; 3 Central Virology Laboratory, Ministry of Health, Chaim Sheba Medical Center, Ramat Gan 5266202, Israel; 4 Infection Prevention & Control Unit, Sheba Medical Center, Ramat Gan 5262504, Israel; 5 Faculty of Medicine, Tel-Aviv University, Tel Aviv 5265601, Israel

**Keywords:** COVID-19, Omicron, influenza, neutralization, hemagglutination

## Abstract

Vaccination against COVID-19 and influenza provides the best defense against morbidity and mortality. Administering both vaccines concurrently may increase vaccination rates and reduce the burden on the healthcare system. This study evaluated the immunogenicity of healthcare workers in Israel who were co-administered with the Omicron BA.4/BA.5 bivalent COVID-19 vaccine and the 2022–2023 quadrivalent influenza vaccine. SARS-CoV-2 neutralizing antibody titers were measured via microneutralization while influenza antibody titers were measured via hemagglutination inhibition. No immunogenic interference was observed by either vaccine when co-administered. Antibody titers against SARS-CoV-2 variants increased significantly in the cohort receiving the COVID-19 vaccine alone and in combination with the influenza vaccine. Antibody titers against the A/H1N1 influenza strain increased significantly in the cohort receiving the influenza vaccine alone and in combination with the COVID-19 vaccine. Antibody titers against B/Victoria increased significantly in the cohort that received both vaccines. This study has important public health implications for the 2023–2024 winter season, and supports co-administration of both vaccines as a viable immunization strategy.

## 1. Introduction

Influenza and coronavirus disease (COVID-19) are acute respiratory diseases with a high burden of morbidity and mortality [1,2]. The most effective strategy for prevention and control of symptomatic influenza and COVID-19 is vaccination [3,4,5,6]. Various types of vaccines have been developed against COVID-19, but waning immune response and breakthrough infections have necessitated the use of booster vaccinations against novel variants, which have been found to be effective at mitigating severe outcomes [7,8]. Influenza vaccines are updated annually to ensure vaccines match current circulating strains [1]. Co-administering influenza and COVID-19 booster vaccines may increase immunization rates and coverage, and alleviate the burden on the healthcare system.

Conflicting results regarding co-administering COVID-19 and influenza vaccines have been published to date. Several sub-studies were performed as part of the clinical trials evaluating first-generation COVID-19 vaccines against the wild-type (WT) strain. Lazarus et al. found that immune responses were not adversely affected with concomitant administration of either ChAdOx1 (adenoviral vector vaccine) or BNT162b2 (mRNA) vaccines with seasonal influenza vaccines, while Toback et al. found that co-administration of the NVX-CoV2373 (protein-based) vaccine with the influenza vaccine resulted in a reduction in antibody response to the NVX-CoV2373 vaccine [9,10]. Izikson et al. evaluated co-administration of a third dose of the mRNA-1273 COVID-19 vaccine with a high dose quadrivalent influenza vaccine in adults aged 65 years and older, and found no evidence of immune interference [11]. Radner et al. reported reduced immunogenicity of the BNT162b2 booster vaccine when administered in combination with the quadrivalent influenza vaccine [12]. Wagenhauser et al. also described reduced anti-SARS-CoV-2 antibody titers in participants receiving a booster dose of either the BNT162b2 or mRNA-1273 vaccines co-administered with a quadrivalent influenza vaccine [13]. Dulfer et al. conducted a single-blind, placebo-controlled randomized clinical trial in individuals ≥60 years to determine the timing and sequence of COVID-19 (BNT162b2 booster) and influenza vaccination (Vaxigrip Tetra). The authors found that the co-administration group was characterized by a lower antibody concentration and reduced neutralizing antibodies against the COVID-19 vaccine [14]. Gonen et al. reported that co-administration of the Omicron BA.4/BA.5 bivalent COVID-19 vaccine and the quadrivalent influenza vaccine resulted in lower antibody levels against COVID-19, but that this was not a substantially inferior immune response [15]. Murdoch et al. conducted a Phase 3, randomized, observer-blind clinical trial in healthy individuals aged 18–64 who had received three previous doses of BNT162b2. The authors found that antibody responses of co-administered COVID-19 (BNT162b2) and a seasonal inactivated influenza vaccine were non-inferior to antibody responses of either vaccine alone [16]. Nearly all the studies detailed above evaluated the immunogenicity of the COVID-19 vaccines via SARS-CoV-2 anti-spike protein immunoglobulin assays, with the exception of Dulfer et al. [14]. Data regarding immunogenicity based on neutralizing antibodies remains scarce.

The objective of this study was to assess if co-administration of the Omicron BA.4/BA.5-adapted bivalent vaccine (second booster) and the quadrivalent influenza vaccine modifies vaccine immunogenicity, specifically by evaluating neutralizing antibodies induced by the bivalent vaccine towards SARS-CoV-2 variants dominant during the 2022–2023 winter season in Israel.

## 2. Materials and Methods

### 2.1. Study Population

A nested study was conducted within an ongoing prospective longitudinal cohort study among healthcare workers (HCWs) at Sheba Medical Center, a tertiary, university-affiliated hospital in Central Israel with 2000 beds and approximately 15,000 HCWs. The cohort includes physicians, nurses or nurse aids, paramedical personnel, administrative or logistic employees, students, volunteers, and retired personnel [17]. The study was approved by Sheba Medical Center’s institutional review board, and all participants provided informed consent. Participants were invited to join the study and provide peripheral blood samples for serological assays prior to the rollout of the first COVID-19 dose in Israel in December 2020, and continued to provide monthly samples in the period following receipt of the second and subsequent vaccine doses. Participants were considered fully vaccinated after receiving two vaccines against COVID-19. In the lead-up to the 2022–2023 winter season, HCWs were encouraged to be vaccinated with the seasonal quadrivalent influenza vaccine (Abbott (Chicago, IL, USA), Influvac Tetra) and the Omicron BA.4/BA.5-adapted bivalent vaccine (Pfizer/BNT). Serum samples were obtained from participants between September 2022 and February 2023. Analysis was conducted on three HCW sub-cohorts. The first cohort included in the analysis had received the seasonal influenza vaccine only. The second cohort had received the COVID-19 vaccine only. The third cohort had received both vaccines concurrently. The cohorts were sex-matched where possible and age-matched within 5 years if an exact match was unavailable.

Antibody titers against four different influenza strains included in the 2022–2023 seasonal influenza vaccine are as follows: A/Victoria/2570/2019 (H1N1), A/Darwin/9/2021 (H3N2), B/Austria/1359417/2021 (Victoria), and B/Phuket/3073/2013 (Yamagata); four different SARS-CoV-2 variants (wild-type (WT), BA.1, BA.5, and BQ.1.1) were tested for each serum sample via hemagglutination inhibition (HI) assay and microneutralization, respectively.

### 2.2. Hemagglutination Inhibition and Microneutralization Assays

The HI assay was performed as previously described [18]. The influenza viral antigens used were from the 2022–2023 WHO influenza reagent kit for the identification of influenza isolates: A/Victoria/2570/2019 (H1N1), A/Delaware/01/2021 (H3N2), B/Michigan/01/2021(Victoria), and B/Phuket/3073/2013 (Yamagata). Serum samples were tested at an initial dilution of 1:20, and up to a final dilution of 1:2560 after RDE treatment and heat inactivation (heated at 56 °C for 30 min). Turkey red blood cells were used, and standardized viral antigen was prepared fresh on the days the assay was performed to a standard dilution of 4 HA units. For geometric mean titer (GMT) calculation, any samples with a negative result by HI assay were assigned a base titer of 1:10. Historical issues have been observed with the ability of H3N2 titers to accurately be measured via HI assay due to a lack of agglutination of avian red blood cells [19]. We are unable to say with certainty whether this affected our assessment of H3N2 antibody titers; however, we do have reliable results from the other three influenza strains.

The microneutralization assay was performed as previously described [20]. The SARS-CoV-2 variants tested were isolated by sequencing nasopharyngeal samples from infected individuals to identify the WT (hCoV19/Israel/CVL-45526-ngs/2020), Omicron BA.1 (hCoV-19/Israel/CVL-n49814/2021), Omicron BA.5 (hCoV-19/Israel/CVL-n51658/2022), and Omicron BQ1.1 (hCoV-19/Israel/CVL-n52292/2023). A median tissue culture infectious dose (100 TCID_50_) of all SARS-CoV-2 variants tested (WT, BA.1, BA.5, and BQ.1.1) was incubated with serially diluted (1:8 to 1:16, 3841) heat-inactivated serum (heated at 56 °C for 30 min) in 96 well plates at 33 °C for 1 h. This combination of virus and serially diluted serum was then inoculated onto VERO-E6 cells and incubated at 33 °C for 5 days, at which point cells were fixed and stained with Gentian violet dye (1%). Neutralizing antibody titer was determined by the highest dilution of sera that presented with no cytopathic effect. For GMT calculation, any samples with a negative result as determined by microneutralization assay were assigned a base titer of 4.

### 2.3. Statistical Analyses

Differences in sex and immunosuppression status by cohort were assessed via chi-square tests. Differences in age between cohorts were assessed via ANOVA. Differences in time between the pre-vaccine sample taken and administration of the vaccine, and the time between the vaccine being administered and the post-vaccine sample being taken were assessed via independent t-test. GMT with 95% CI was used to assess the means of antibody titers reported for each participant for each strain or variant. Differences between pre-vaccination and post-vaccination antibody titers were assessed using the Wilcoxon Rank Sum Test. Fold change was assessed as the geometric mean ratio (GMR), which was calculated as the mean of the ratio of antibody titers reported for each participant (post/pre) for each strain or variant. All statistical analyses were performed in SAS Studio 9.04.01. Graphics were created in GraphPad Prism 9.5.1.

## 3. Results

A total of 54 HCWs were included in the study, with each cohort comprising 18 individuals. Study population characteristics are presented in Table 1. The mean age was 65 years, almost 80% were female, and most had no history of immunosuppression.

All participants were fully vaccinated against COVID-19. Of the cohort of 18 that received the COVID-19 vaccine alone, 4 (22%) received one booster dose, and 11 (61%) received two booster doses. Of the cohort of 18 that received concurrent influenza and COVID-19 vaccines, 5 (27%) received one booster dose and 13 (72%) received two booster doses. There were no statistically significant differences between these groups. Pre- and post-vaccination samples were taken an average of 4.5 days prior and 36 days after, respectively.

**Figure 1:**
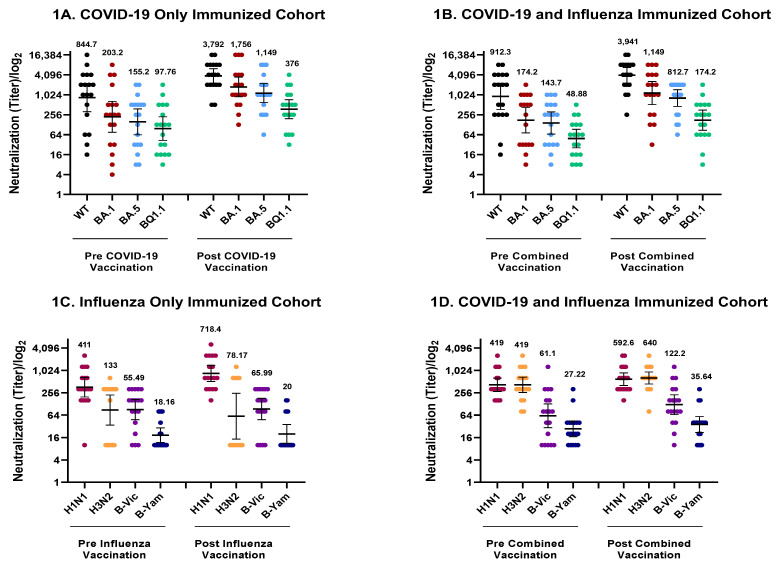
Comparison of antibody titers before and after vaccination with influenza strain and COVID-19 variant, Israel (September 2022–February 2023). (**A**,**B**) show neutralizing antibody titers against the WT, BA.1, BA.5, and BQ1.1 SARS-CoV-2 variants before and after vaccination. Figure 1A shows antibody titers among those who received only the COVID-19 vaccine. (**B**) shows antibody titers among those who received both the COVID-19 and the influenza vaccine. (**C**,**D**) show hemagglutinating antibody titers against influenza strains A/H1N1, A/H3N2, B/Victoria, and B/Yamagata before and after vaccination. (**C**) shows antibody titers among those who received only the influenza vaccine. (**D**) shows antibody titers among those who received both the influenza vaccine and the COVID-19 vaccine. Numbers over each strain indicate geometric mean titers. Whiskers indicate 95% confidence intervals.

**Figure 2:**
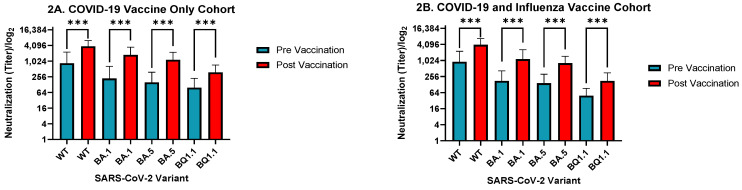

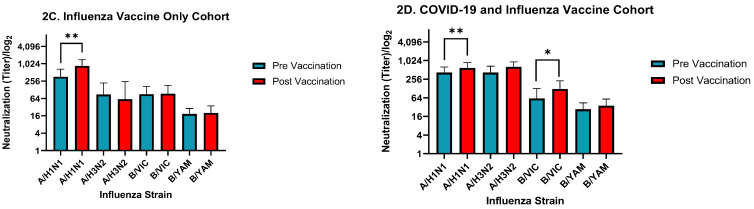
Statistical significance of changes in antibody titers, before and after vaccination, caused by influenza strain and COVID-19 variant, Israel (September 2022–February 2023). (**A**) shows the statistical significance of COVID-19 microneutralization titers for those who received only the COVID-19 vaccine. (**B**) shows the statistical significance of COVID-19 microneutralization titers for those who received both vaccines. (**C**) shows the statistical significance of hemagglutination titers for those who received only the influenza vaccine. (**D**) shows the statistical significance of hemagglutination titers for those who received both vaccines. * refers to *p*-value < 0.05, ** refers to *p*-value < 0.01, *** refers to *p*-value < 0.001.

Neutralizing antibody titers against all SARS-CoV-2 variants increased significantly in both the cohorts that received the COVID-19 vaccine only, and in combination with the influenza vaccine (Figure 1A,B and Figure 2A,B). In the COVID-19 vaccine-only cohort, *p*-values were reported as the following: WT = 0.0002; BA.1 < 0.0001; BA.5 < 0.0001; and BQ1.1 = 0.0002. In the combination cohort, *p*-values were reported as follows: WT ≤ 0.0001; BA.1 < 0.0001; BA.5 < 0.0001; and BQ1.1 ≤ 0.0001. Additional details on GMT values are provided in Supplementary Table S1. The mean fold change increase of the WT virus and Omicron BA.1, BA.5 and BQ1.1 variants were 16.61, 19, 13.22, and 8.72, respectively, in those who received the COVID-19 vaccine only, and 9.11, 15.33, 11, and 10.83, respectively, in those who received both vaccines. Additional details on mean fold change values are provided in Supplementary Table S2.

Antibody titers against A/H1N1 increased significantly (*p* = 0.0027) in the influenza vaccine cohort (Figure 1C and Figure 2C) with a mean fold change increase of 2.42. Antibody titers against the A/H1N1 strain and B/Victoria lineage increased significantly (*p* = 0.0098 and *p* = 0.0269, respectively) in the combination cohort (Figure 1D and Figure 2D). The mean fold change increase of A/H1N1 and B/Victoria in this cohort were 1.58 and 2.81, respectively.

There was no statistically significant difference in the fold change in antibody titer when the COVID-19 or influenza vaccines were administered alone, or when both vaccines were co-administered.

## 4. Discussion

Our study found no significant differences in immunogenicity when the 2022–2023 seasonal quadrivalent influenza vaccine and the Omicron BA.4/BA.5-adapted bivalent vaccine were co-administered. To our knowledge, this is the first study assessing the neutralizing capacity of the COVID-19 vaccine and currently circulating variants when co-administered with the 2022–2023 quadrivalent influenza vaccine. Neutralization using the current vaccine and circulating variants offers a better understanding of the immunogenicity of the Omicron BA.4/BA.5-adapted bivalent COVID-19 vaccine and allows us to fully evaluate if immune interference occurs when a quadrivalent influenza vaccine is administered simultaneously.

This study supports co-administration of the seasonal quadrivalent influenza and COVID-19 booster vaccines. Co-administration can streamline vaccine delivery by reducing the frequency of encounters with the healthcare system, which may alleviate the burden on healthcare personnel, and can also improve immunization rates. A meta-analysis examining combined influenza and COVID-19 booster vaccines found that combining both vaccines can increase the uptake of COVID-19 boosters by over 56% [21].

Our findings are consistent with Lazarus et al. [9], Izikson et al. [11], Gonen et al. [15], and Murdoch et al. [16] who found that immunogenicity was not attenuated when both the COVID-19 and seasonal influenza vaccines were administered together. Our findings contrast with Toback et al., Radner et al., and Wagenhauser et al., who describe reduced immunogenicity of the COVID-19 vaccine when co-administered with the influenza vaccine [10,12,13]. However, these studies assessed changes to antibody levels targeting the SARS-CoV-2 WT virus spike protein, while our study evaluated the neutralizing capacity of sera antibodies regardless of epitope. Additionally, neutralization better captures the immunological impact of the vaccine compared with measuring antibody levels. Our results also differ from Dulfer et al. [14] who reported significantly lower neutralizing antibody levels against the Delta and Omicron BA.1 SARS-CoV-2 variants in the co-administration group, compared to a reference group that received a COVID-19 booster vaccination alone. Important differences between both studies include the makeup of the study cohorts, and the type of vaccine administered. Our study cohort included participants under the age of 60 years and was made up of relatively healthy HCWs, the majority of whom had received one or two booster vaccines—all mRNA vaccines. The study cohort in Dulfer et al. [14] was composed of individuals ≥60 years who had not yet received booster vaccination, and with a heterogenous COVID-19 vaccine history including adenoviral vector or mRNA vaccines. Additionally, the COVID-19 vaccine administered in our study was the Omicron BA.4/BA.5-adapted bivalent vaccine (Pfizer/BNT), whilst the COVID-19 vaccine administered in Dulfer et al.’s study [14] was the BNT162b2 vaccine against the WT strain. Previous studies have found that the WT BNT162b2 vaccine does not neutralize the Omicron strain as efficiently as previous strains [22,23,24]. The Omicron BA.4/BA.5 vaccine was adapted to better match and provide broader protection against circulating Omicron variants.

While the current study did not address reactogenicity, previous studies analyzed safety profiles. Most studies found similar reactogenicity profiles between the COVID-19 and co-administration groups [9,11,12,14,15,16]. Several studies found lower rates of reactogenicity in the influenza-only arm [11,12], while one study found that reactogenicity events were slightly more common in the co-administration group [10] and another found that side effects were less common in the co-administration group [13]. Overall co-administration of both the influenza and COVID-19 vaccines was found to be safe and well tolerated.

Poor immunogenicity to the 2022–2023 seasonal quadrivalent influenza vaccine was observed in our study. This may be due to the fact that older people experience considerably lower serological responses to influenza vaccination compared with younger adults [25,26]. The average age of our HCW cohort was 65, which may have affected the level of antibodies developed. Further research is required to evaluate the immunogenicity of currently available influenza vaccines.

Our study had several limitations. Our cohort of HCWs was mostly healthy and therefore our results may not be representative of the general population [17]. Secondly, we were only able to analyze two time points for each participant, and therefore could not observe longer-term trends in immunogenicity. Lastly, the influenza vaccine administered in our study was the quadrivalent inactivated vaccine. Further research is required to determine if our findings apply to live, attenuated, or high-dose vaccines.

## 5. Conclusions

Our study evaluated neutralizing antibodies of the Omicron BA.4/BA.5-adapted bivalent COVID-19 vaccine against variants circulating during the 2022–2023 winter season in Israel co-administered with the quadrivalent influenza vaccine in healthcare workers. Our study found that co-administration did not attenuate the immunogenicity of either vaccine. These findings support the co-administration of both vaccines, which can inform public health strategies and vaccination policies for the upcoming 2023–2024 winter season.

## Figures and Tables

**Table 1:** Baseline characteristics of the study population. Results are displayed as mean (standard deviation).

		All	Received Influenza Vaccine Only	Received COVID-19 Vaccine Only	Received Both Vaccines	*p*-Value
		n = 54	n = 18	n = 18	n = 18	
Age (years)		65.20 (9.74)	64.22 (9.81)	65.94 (9.31)	65.44 (10.55)	0.8661
Gender (female)		79.63%	77.78%	83.33%	77.78%	0.8921
Immunosuppression status	None	90.74%	100%	77.78%	94.44%	0.0570
	Unknown	9.26%	0	22.22%	5.56%	
Days between vaccine and pre-sample (Influenza)		5.28 (7.72)	6.83 (7.98)	NA	3.72 (7.34)	0.2320
Days between vaccine and pre-sample (COVID-19)		3.86 (6.72)	NA	4.00 (6.35)	3.72 (7.34)	0.2800
Days between vaccine and post-sample (Influenza)		36.39 (5.21)	38.00 (5.14)	NA	34.78 (4.88)	0.0623
Days between vaccine and post-sample (COVID-19)		35.94 (4.71)	NA	37.11 (4.36)	34.78 (4.88)	0.1401

## Data Availability

The data presented in this study are available on request from the corresponding author.

## Supplementary Materials

The following supporting information can be downloaded at: https://www.mdpi.com/article/10.3390/vaccines11101624/s1, Table S1: GMT and CI related to Figure 1 & Figure 2; Table S2: GMR (fold change) and CI.
