# Supplementary material for: Immunogenicity of Co-Administered Omicron BA.4/BA.5 Bivalent COVID-19 and Quadrivalent Seasonal Influenza Vaccines in Israel during the 2022–2023 Winter Season
Source: Vaccines (Basel). 2023 Oct 22;11(10):1624. doi: 10.3390/vaccines11101624 (PMC10610948; doi:10.3390/vaccines11101624)
Supplement: Supplementary file 1 [file vaccines-11-01624-s001.zip › Supplementary Table S1.pdf]

Supplementary Table S1: GMT and CI related to Figure 1 & Figure 2

For these analyses, the mean differences between cohorts were calculated for each participant; the means of the individual values are presented here.

		Influenza vaccine cohort			COVID vaccine cohort			Co-administered vaccines cohort		
		**Pre**	**Post**	**p-value**	**Pre**	**Post**	**p-value**	**Pre**	**Post**	**p-value**
**Microneutralization assay WT**	Geometric mean	2389	2048	0.5313	844.7	3792	0.0002	912.3	3941	<0.0001
	95% Confidence interval	(1346, 4241)	(1096, 3829)		(316.8, 2252)	(2300, 6253)		(370.0, 2249)	(2308, 6729)	
**Microneutralization assay Omicron BA.1**	Geometric mean	670.4	597.3	0.2227	219.5	1756	<0.0001	174.2	1149	<0.0001
	95% Confidence interval	(314.1, 1431)	(307.4, 1160)		(74.96, 642.5)	(885.1, 3482)		(72.35, 419.3)	(514.4, 2568)	
**Microneutralization assay Omicron BA.5**	Geometric mean	438.9	438.9	0.625	155.2	1149	<0.0001	143.7	812.7	<0.0001
	95% Confidence interval	(206.7, 931.9)	(219.0, 879.4)		(63.16, 381.3)	(593.5, 2226)		(66.62, 309.8)	(455.4, 1450)	
**Microneutralization assay Omicron BQ 1.1**	Geometric mean	128	128	0.625	97.76	376	0.0002	48.88	174.2	<0.0001
	95% Confidence interval	(64.90, 252.4)	(71.72, 228.4)		(43.04, 222.0)	(194.3, 728.4)		(25.82, 92.53)	(86.35, 351.3)	
**Hemagglutination Inhibition Assay H1N1**	Geometric mean	411	718.4	0.0027	615.8	640	0.5625	419	592.6	0.0098
	95% Confidence interval	(275.5, 613.2)	(522.5, 987.7)		(498.1, 761.4)	(517.1, 792.0)		(277.5, 632.6)	(401.1, 875.3)	
**Hemagglutination Inhibition Assay H3N2**	Geometric mean	133	78.17	0.4219	746.6	640	0.3125	419	640	0.1426
	95% Confidence interval	(50.14, 352.8)	(21.77, 280.6)		(444.8, 1253)	(374.4, 1094)		(260.6, 673.7)	(440.4, 930.1)	
**Hemagglutination Inhibition Assay B/Victoria**	Geometric mean	55.49	65.99	0.2092	42.38	24.72	0.001	61.1	122.2	0.0269
	95% Confidence interval	(31.24, 98.57)	(33.88, 128.5)		(18.59, 96.60)	(12.10, 50.51)		(29.16, 128.0)	(66.00, 226.2)	
**Hemagglutination Inhibition Assay B/Yamagata**	Geometric mean	18.16	20	0.6563	19.62	15.27	0.125	27.22	35.64	0.127
	95% Confidence interval	(12.17, 27.10)	(11.60, 34.49)		(12.16, 31.65)	(10.29, 22.66)		(16.90, 43.82)	(21.81, 58.23)	
