# Supplementary material for: Immunogenicity of Co-Administered Omicron BA.4/BA.5 Bivalent COVID-19 and Quadrivalent Seasonal Influenza Vaccines in Israel during the 2022–2023 Winter Season
Source: Vaccines (Basel). 2023 Oct 22;11(10):1624. doi: 10.3390/vaccines11101624 (PMC10610948; doi:10.3390/vaccines11101624)
Supplement: Supplementary file 1 [file vaccines-11-01624-s001.zip › Supplementary Table S2.pdf]

Supplementary Table S2: GMR (fold change) and CI

Comparison of fold-change in antibody titers in sera from healthcare workers (n=54) when either the Omicron BA.4/BA.5 adapted bivalent vaccine or quadrivalent seasonal 2022-2023 influenza vaccine were administered alone or both vaccines were co-administered. For these analyses, the GMR was calculated for each participant; the means of the individual values are presented here.

	**COVID-19 Vaccine**		**Both Vaccines**		**Influenza Vaccine**		**p-value**
	**GMR**	**95% CI**	**GMR**	**95% CI**	**GMR**	**95% CI**	
**SARS-CoV-2 variants:**							
**WT**	16.61	(0.52, 32.70)	9.11	(1.36, 16.86)	NA	NA	0.67
**BA.1**	19.00	(7.38, 30.62)	15.33	(0.58, 30.09)	NA	NA	0.71
**BA.5**	13.22	(5.33, 21.11)	11.00	(2.93, 19.07)	NA	NA	0.48
**BQ.1.1**	8.72	(1.35, 16.10)	10.83	(0.00, 25.49)	NA	NA	0.91
**Influenza strains:**							
**A/H1N1**	NA	NA	1.58	(1.18, 1.99)	2.42	(1.05, 3.78)	0.56
**A/H3N2**	NA	NA	1.89	(1.26, 2.52)	5.71	(0.00, 14.71)	0.30
**B/Victoria**	NA	NA	2.81	(1.53, 4.09)	1.88	(0.85, 2.91)	0.09
**B/Yamagata**	NA	NA	1.58	(1.06, 2.10)	2.73	(0.00, 5.49)	0.24
